# Supplementary material for: Single Nucleotide Polymorphisms in MIR143 Contribute to Protection against Non-Hodgkin Lymphoma (NHL) in Caucasian Populations
Source: Genes (Basel). 2019 Feb 27;10(3):185. doi: 10.3390/genes10030185 (PMC6471575; doi:10.3390/genes10030185)
Supplement: Supplementary file 1 [file genes-10-00185-s001.pdf]

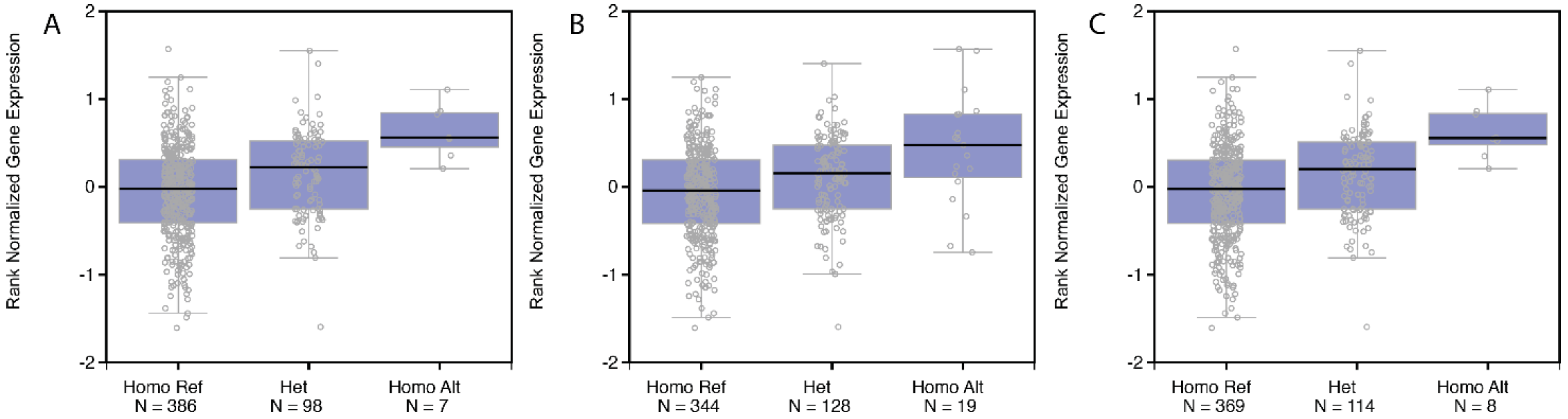

Figure S1. **Significant eQTLs for 3 analysed *MIR143* SNPs**. a) rs17723799, b) rs3733846 and c) rs41291957 in 491 subjects showing increased *MIR143* host gene expression with heterozygous (Het) and homozygous alternate (Homo Alt) genotypes in skeletal muscle (GTEx Portal).
